# A New Thermophilic Nitrilase from an Antarctic Hyperthermophilic Microorganism

**DOI:** 10.3389/fbioe.2016.00005

**Published:** 2016-02-29

**Authors:** Geraldine V. Dennett, Jenny M. Blamey

**Affiliations:** ^1^Fundación Científica y Cultural Biociencia, Santiago, Chile; ^2^Doctorado en Biotecnología, Universidad de Santiago, Santiago, Chile

**Keywords:** cyanidase, N-glycosylation, thermostable, nitriles, Antarctica

## Abstract

Several environmental samples from Antarctica were collected and enriched to search for microorganisms with nitrilase activity. A new thermostable nitrilase from a novel hyperthermophilic archaea *Pyrococcus sp*. M24D13 was purified and characterized. The activity of this enzyme increased as the temperatures rise from 70 up to 85°C. Its optimal activity occurred at 85°C and pH 7.5. This new enzyme shows a remarkable resistance to thermal inactivation retaining more than 50% of its activity even after 8 h of incubation at 85°C. In addition, this nitrilase is highly versatile demonstrating activity toward different substrates, such as benzonitrile (60 mM, aromatic nitrile) and butyronitrile (60 mM, aliphatic nitrile), with a specific activity of 3286.7 U mg^−1^ of protein and 4008.2 U mg^−1^ of protein, respectively. Moreover the enzyme NitM24D13 also presents cyanidase activity. The apparent Michaelis–Menten constant (*K*_m_) and *V*_máx_ of this Nitrilase for benzonitrile were 0.3 mM and 333.3 μM min^−1^, respectively, and the specificity constant (*k*_cat_/*K*_m_) for benzonitrile was 2.05 × 10^5^ s^−1^ M^−1^.

## Introduction

Chemical and pharmaceutical industries use a diversity of nitriles as feedstock material for the synthesis of a wide range of compounds, drug intermediates, pesticides (such as bromoxynil and dichlobenil), polymers and even solvents, such as acetonitrile (Jallageas et al., 1980; Cowan, et al., 1998; Prasad et al., 2007; DeSantis and DiCosimo, 2009). Nitriles are organic compounds containing in its structure a cyano group (-CN). They are transformed to carboxylic acids by several chemical processes, but these processes typically require strong acidic or basic conditions, high temperatures and usually produce unwanted byproducts and a lot of inorganic waste (Dash et al., 2009). This is not the case when this transformation is biocatalyzed by nitrilases making the transformation in a single step obtaining the respective carboxylic acid.

Nitriles (organic cyanide) are widely distributed in nature but most of them are chemically synthesized (Banerjee et al., 2002; Gong et al., 2012). Microorganism as well as plants are able to transform nitriles to the corresponding carboxylic acids and amides. These reactions are catalyzed by two different enzymes, nitrilase (EC 3.5.5.1) and nitrile hydratase (EC 4.2.1.84) (Arnaud et al., 1976).

In soil–water systems, the bioavailability and solubility of cyanide are also determining factors (Aronstein et al., 1994; Dash et al., 2006). Cyanide biodegradation can be carried out by more than one pathway in some organisms (Raybuck, 1992; Ezzi-Mufaddal and Lynch James, 2002; Akcil et al., 2003; Ebbs, 2004). Conditions such as oxygen, pH, and cyanide concentration determined the metabolic pathway that will be involved in the degradation. Enzymes such as cyanide hydratase, forming formamide (HCN + H_2_O → HCONH_2_), or cyanidase, which produces formate and ammonia (HCN + 2H_2_O → HCOOH + NH_3_) (Ebbs, 2004) are involved in cyanide biodegradation.

Nitrilases are enzymes that catalyze the direct conversion of nitriles to their respective carboxylic acid with liberation of ammonia, while nitrile hydratase catalyze the formation of amides from nitriles.

Enzymatic biotransformation of nitriles has been considered an efficient alternative route with respect to chemical methods in industry, providing a very valuable alternative for efficiency, speed, and environmental friendliness. A great variety of nitrilases and nitrile hydratases and their applications in industry have been extensively studied (Arnaud et al., 1976; Mylerova and Martinkova, 2003; DeSantis and DiCosimo, 2009; Gong et al., 2012) for the production of amides and organic acid highly valued in industry (Ramakrishna et al., 1999) To perform the chemical synthesis of different compounds at elevated temperatures has many advantages for the industry, transfer rates improvement, substrate solubility enhancement, decrement in the viscosity of the solutions, and reducing the risk of contamination (Egorova and Antranikian, 2005). The relative instability of mesophilic nitrilases makes them to become inactivated at temperatures above 50°C (Harper, 1985; Kobayashi et al., 1990; Gong et al., 2012). For that reason, the interest in enzymes from thermophilic and hyperthermophilic microorganisms has increased and the fact that these proteins are resistant to chemical and physical denaturation and to proteolysis (Daniel et al., 1982; Egorova and Antranikian, 2005). Only two moderately thermo active nitrilases have been described so far. One of them was isolated from *Acidovorax facilis* 72W (Chauhan et al., 2003) and the second was isolated from *Bacillus pallidus* Dac521. This thermo active nitrilase has an optimum temperature of 65°C; however, after 13-min exposure to 70°C it is inactive (Almatawah et al., 1999). One thermostable recombinant nitrilase was isolated from *Pyrococcus abyssi* (Mueller et al., 2006). This nitrilase catalyzes the transformation of aliphatic nitriles mainly but inactive at concentrations above 12 mM of substrate malononitrile.

The growing need of new nitrile-degrading enzymes (Mathew et al., 1988; Wyatt and Linton, 1988) and the enzymatic detoxification of a nitrile-based herbicides (Harper, 1985; Stalker et al., 1988) along with the instability of mesophilic nitrile-metabolizing enzymes (Nagasawa et al., 1990) led us to investigate hyperthermophilic microorganisms as an alternative source of these activities (Cramp et al., 1997; Pereira et al., 1998; Mueller et al., 2006). In this work, we describe the purification and characterization of a new thermoactive nitrilase from the hyperthermophilic anaerobic archaeon *Pyrococcus* sp. M24D13 that it is able to use inorganic cyanides as substrate.

## Materials and Methods

### Isolation of Microorganism and Culture Condition

Soil samples were collected from Fumarole Bay in Deception Island, Antarctica during the scientific expedition ECA 46. The nitrile-degrading microorganisms were isolated from these soil samples by serial dilutions using medium described by Mueller et al., 2006 containing nitriles.

The isolation of the microorganism was carried out under strict anaerobic conditions and has been identified through the analysis of its 16S rRNA gene complete sequence.

The microorganism M24D13 was cultivated anaerobically at 95°C in the following medium (pH 7.0) containing (per liter): 23.4 g NaCl, 10.8 g MgCl_2_ × 6H_2_O, 4.0 g Na_2_SO_4_, 0.7 g KCl, 0.2 g NaHCO_3_, 0.2 g CaCl_2_ × 2H_2_O, 0.09 g KBr, 0.025 g SrCl_2_ × 6H_2_O, 0.025 g H_3_BO_3_, 0.003 g NaF, 5 g elementary sulfur, 1 g yeast extract, 4 g peptone, 0.35 g KH_2_PO_4_, 0.7 g NH_4_Cl, and 10 ml trace element solution according to Balch et al. (1979).

### Phylogenetic Analysis of the 16S rRNA Gene Sequence and the Nitrilase NitM24D13 Sequence

Genomic DNA of the isolate was extracted using a phenol–chloroform protocol. The genomic DNA of the isolated microorganism was completely sequenced in Georgia Genomics Facility (GGF, University of Georgia, USA) obtaining from it the complete sequence of the 16S rRNA gene (GenBank, accession number SUB1008503 Pyrococcus_sp_M24D13 KT267175) and the NitM24D13 gene. Related sequences were obtained from GenBank database [National Center for Biotechnology Information (NCBI), Bethesda, MD, USA] PDB, SwissProt, PIR, and PRF using the BLAST search program. The sequences were aligned using multiple sequence alignment software, CLUSTAL W ver. 1.81. A phylogenetic tree was constructed with MEGA 5 software (Tamura et al., 2011) based on the information of complete 16S rRNA sequences of 13 strains similar to *Pyrococcus sp*. M24D13, using the method of *neighbor-joining* (Saitpu and Nei, 1987) with a bootstrap analysis of 1000 replicates.

### Protein Assay

Protein was determined using the Bio-Rad Bradford kit according to Bradford (1976). Bovine serum albumin was used as standard.

### Enzyme Assay

Nitrilase activity of the microbial enzyme was assayed by measuring the production of NH_3_ during the hydrolysis of benzonitrile to benzoic acid by the method of Fawcett and Scott (1960). The standard assay was performed in duplicate at 80°C in tubes containing 0.9 ml of 30 mM benzonitrile in 100 mM phosphate buffer pH 8.0 with 2 mM EDTA, to which was added 0.1 ml of crude extract or purified protein. Mixtures were incubated for 5 min, after which time the reaction was terminated by the addition of 330 mM sodium phenoxide (1 ml), followed by 0.01% sodium nitroprusside (1 ml) and 20 mM sodium hypochlorite (1 ml). The assay mixture was thoroughly shaken, heated for 10 min at 95°C to allow color development, then diluted with water (6 ml), and the enzymatic activity was measured at an absorbance of 640 nm in a Shimadzu UV-VIS spectrophotometer.

For the measurement of cyanidase activity, potassium cyanide (KCN) was used as substrate replacing benzonitrilo to detect cyanidase in the standard assay shown above.

### Purification of the Nitrilase from *Pyrococcus* sp. M24D13

All columns used in the protein purification were controlled by a Pharmacia FPLC (fast performance liquid chromatography) system.

#### Step I: Preparation of Cell-Free Extract

A cellular disruption method was specially designed for hyperthermophilic microorganisms. Washed cells (90 g) from 40 l of culture medium were suspended in 800 ml of 50 mM Tris–HCl buffer pH 7.0 containing 2 mM EDTA and lysozyme (3 mg ml^−1^) and were incubated at 37°C for 60 min. Triton X100 was then added to a concentration of 4% v/v. The cell suspension was homogenized and incubated 15 min at 37°C. Then, it was disrupted by sonication for a total duration of 4 h using a sonicator bath (Branson sonifier 450). Cell debris was removed by centrifugation (17,968 × *g* for 40 min) and the supernatant solution was used as the crude extract for the purification. The crude extract was concentrated by ultrafiltration with Amicon cellulose membrane (M^r^ Cut-off 10,000).

#### Step II: Ammonium Sulfate Fractionation

Solid ammonium sulfate was added to the crude extract to give 55% saturation. After being stirred for 1 h, the suspension was centrifuged (17,968 × *g* for 20 min at 4°C), and the pellet was dissolved in Tris–HCl buffer (50 mM, pH 8.0), containing 2 mM EDTA (buffer A).

#### Step III: Hydrophobic Interaction Chromatography

After step II, ammonium sulfate was added to the protein solution in small portions with stirring to bring the solution to 1.6 M saturation. The enzyme solution was loaded onto a Octyl Sepharose column (GE Healthcare Life Sciences) pre-equilibrated with buffer A, containing 1.6 M (NH_4_)_2_SO_4_. Bound proteins were eluted with a decreasing salt gradient (from 1 to 0 M) of (NH_4_)_2_SO_4_ in buffer A, at a flow rate of 1 ml min^−1^.

#### Step IV: Size Exclusion Chromatography

Active fractions were pooled, concentrated to a volume of 0.5 ml by ultrafiltration using an Amicon cellulose membrane (M^r^ cut-off 10,000), applied to a column (GE Healthcare, Tricorn 10/600) of Superdex-200 (Pharmacia Biotech), equilibrated with buffer A containing 0.2 M NaCl and eluted with the same buffer at a flow rate of 0.7 ml min^−1^. The fractions with nitrilase activity were concentrated by ultrafiltration (PM-10 membrane filter; Amicon), and were stored at 4°C.

#### Step V: Ion Exchange Chromatography

The concentrated protein solution after size exclusion chromatography was applied to a 1 ml Q-HiTrap HP (GE Healthcare Life Sciences) anion exchange column. After loading the protein solution, the column was washed with buffer A until there was no further elution of protein. The enzyme was then eluted with a linear gradient of NaCl (from 0 to 1 M) in the same buffer for 90 min.

Protein fractions containing the nitrilase activity were analyzed by SDS-PAGE (polyacrylamide gel electrophoresis) according to Laemmli (1970) in 15% polyacrylamide gels with a Tris–Glycine buffer system.

### Molecular Mass Determination

The apparent molecular mass of the native nitrilase was estimated by gel filtration chromatography on a column (GE Healthcare, Tricorn 10/600) of Superdex-200 (Pharmacia Biotech) equilibrated with buffer A containing 0.2 M NaCl and calibrated using urease 547 kDa, bovine glutamate dehydrogenase 300 kDa, bovine serum albumin 66 kDa, egg ovalbumin 45 kDa, and lysozyme 14.3 kDa as standard proteins. The void volume was determined using Blue dextran (2000 kDa). The enzyme relative molecular mass value under non-denaturating condition was determined from the semi-log plot of the standard protein molecular mass against *K*_av_ values. The subunit molecular mass of nitrilase was determined by SDS-PAGE (15%) according to the method of Laemmli (1970) using a BenchMark™ pre-stained protein ladder. SDS gel was stained for proteins with a silver stain protocol based on the method of Sammons et al. (1981).

### Thermostability

For determination of nitrilase thermostability, the enzyme was placed in small tubes with O-ring-sealed caps and incubated for 14 h in a dry bath (Major Science, MD-02N-220) at 85°C. Samples were taken every hour and assayed for enzyme activity. Residual activity was determined at 80°C under the conditions described in Section “Enzyme Assay.”

### Effect of Metal Ions and Other Reagents

The effect of various reagents and salts of different metal ions (Ag^+^ and Hg^2+^) on the enzymatic activity was tested by pre-incubating the enzyme with these compounds in 100 mM phosphate buffer (pH 7.5) at room temperature for 5 min at a final concentration of 1–5 mM. Then, 3 mM benzonitrile was added and the standard activity was measured as described above.

## Results

### Phylogenetic Analysis of the 16S rRNA Gene Sequence of M24D13

**Figure 1:**
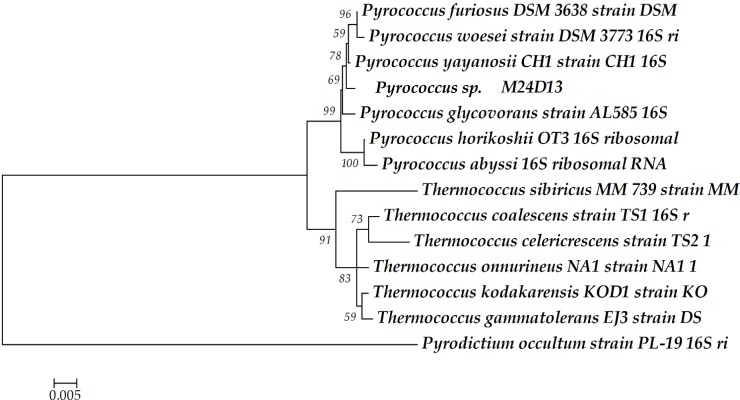
**Phylogenetic tree of the 16S rRNA gene sequence**. The sequence obtained from the isolated microorganism M24D13 was compared with date base sequences available in GenBank to the Archaea domain, using *Pyrodictium occultum* as outgroup. The phylogenetic tree was made by Neighbor-joining method with 1000 bootstrap replicates.

The phylogenetic analysis of complete 16S rRNA sequence of the isolated microorganism M24D13 shows that this microorganism belongs to the genus *Pyrococcus* and is related to the species *yayanossi*, as shown in the phylogenetic three (Figure 1).

### Analysis of the Amino Acid Sequence of the Nitrilase NitM24D13 Gene

**Figure 2:**
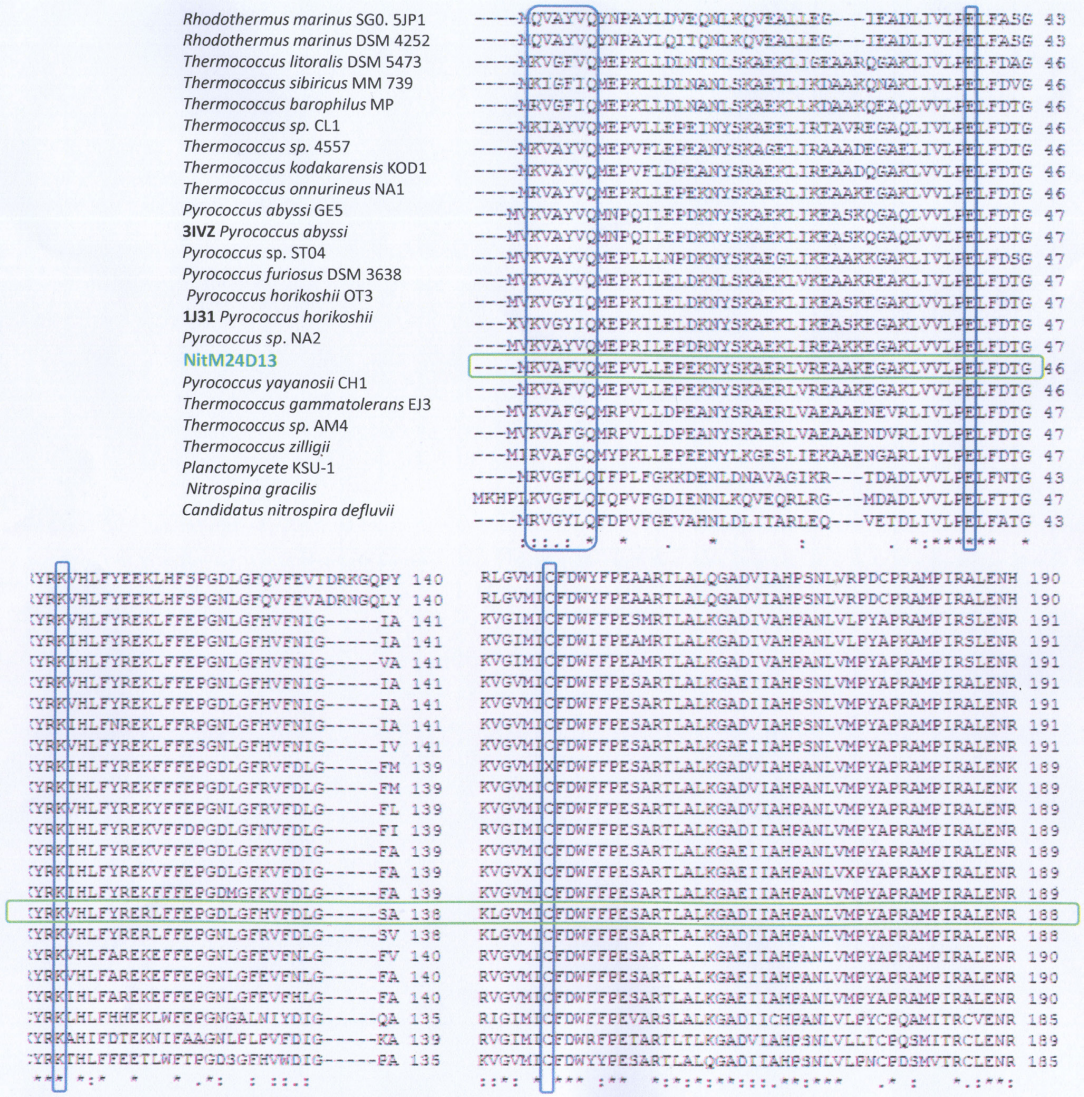
**Analysis of the amino acid sequence alignment of the nitrilase gene of M24D13 microorganism against GenBank, PDB, SwissProt, PIR, and PRF data bases using Clustal W software**. The red marked columns indicate the amino acids that belong to the catalytic triad of the known nitrilase enzymes (E-K-C). The blue marked column corresponds to the consensus sequence present at the N-terminal for nitrilases. The green marked lane corresponds to the sequence of NitM24D13.

The complete amino acid sequence of the putative nitrilase present in the M24D13 genome was analyzed to verify that this sequence corresponds to a nitrilase. The conserved regions were detected at the positions of amino acids 41(E), 112(K), and 145(C). The cysteine residue (145 amino acid), which is involved in the currently known catalytic mechanism (Pace and Brenner, 2001) is absolutely conserved in all compared sequences. Additionally, the N-terminal signature of already described microbial nitrilases KVA-x-VQ (Stalker et al., 1988; Hoyle et al., 1998; Mueller et al., 2006; Gong et al., 2012) was also identified in the sequence of nitrilase from the archaeal genome M24D13 (Figure 2). The results of the analyzed sequence allowed us to confirm that the examined gene corresponds to nitrilase.

### Nitrilase Purification

**Figure 3:**
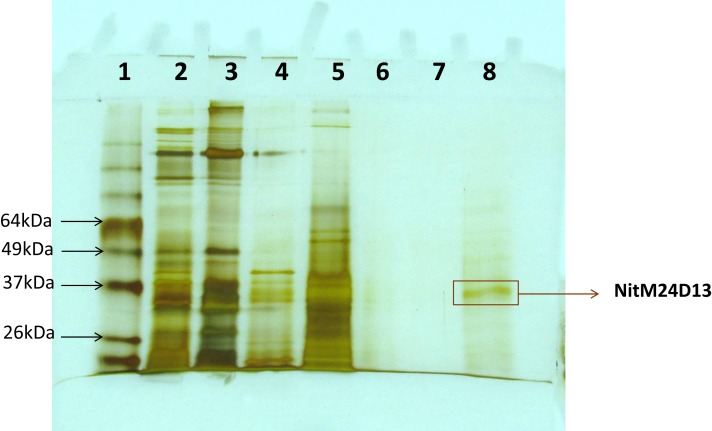
**SDS-PAGE (15%) gel electrophoresis of nitrilase fractions obtained from the purification protocol, stained with silver nitrate**. The band marked on the gel corresponds to the band of interest. Lane 1, Bench Mark^TM^ molecular weight marker (Invitrogen). Lane 2, Crude extract. Lane 3, concentrated crude extract. Lane 4, protein supernatant precipitated with 55% (NH_4_)_2_SO_4_. Lane 5, fraction 10 from Octyl-Sepharose chromatography. Lanes 6 and 7 correspond to fractions 4 and 5 from Superdex-200 chromatography. Lane 8, fraction 4 from Q-HiTrap chromatography.

A new nitrilase activity was purified to near homogeneity from benzonitrile-induced cells as described in methods using a combination of ammonium sulfate fractionation, hydrophobic interaction, gel filtration, and ion exchange chromatography. The nitrilase from *Pyrococcus sp*. M24D13 (NitM24D13) was purified 219-fold with a yield of approximately 4.7% from the cell-free extract using benzonitrile as substrate (Table 1) and SDS-PAGE analysis of fractions from the complete protocol are shown in Figure 3.

**Table 1:** **Summary of the purification protocol for nitrilase from M24D13**.

Samples	Total activity (U)	U/ml	Total protein (mg)	Specific activity (U mg^**−**1^)	Volume (ml)	Yield (%)	Purification (fold)
Crude cell extract	4975.0	6.7	331.5	15.0	740	100	1
Ultrafiltrate crude cell extract (10 kDa)	3931.4	21.8	72.5	54.2	180	79	3.6
55% (NH_4_)_2_SO_4_ fraction (p.p)	10,521.4	56.7	17.6	598.4	185.5	212	39.9
Octyl-Sepharose	5801.7	252.3	2.3	2533.2	23	117	168.8
Superdex-200	4388.9	319.2	1.7	2576.1	13.8	88	171.7
Q-Hi Trap HP	234.1	117.0	0.1	3286.7	2	5	219.0

### Molecular Mass Determination

The apparent molecular mass of the native nitrilase under non-denaturating condition was estimated in 38.5 kDa and the enzyme relative molecular mass of the subunit under denaturating condition was determine in 37 kDa.

### Effect of Temperature, pH, and Thermostability

The influence of temperature on the specific activity was determined under standard assay conditions. The effect of pH on specific activity was measured as previously described at various pH values between 4.0 and 9.5 in EPPS (Sigma), CAPS (Sigma), phosphate (Winkler), and citrate (Winkler) buffers at the optimal temperature.

**Figure 4:**
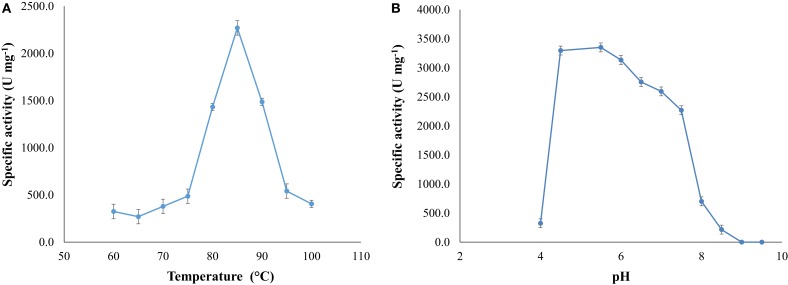
**(A)** Optimal temperature for NitM24D13 nitrilase activity and **(B)** optimal pH for the NitM24D13 nitrilase activity. The error bars represent the SD of three independent experimental replicates.

Optimal activity occurred at temperature of 85°C (at pH 7.5) and at pH around 5.5 (at 85°C) (Figure 4).The activity of the enzyme increased as the temperature changed from 70 to 85°C, diminishing at higher temperatures.

**Figure 5:**
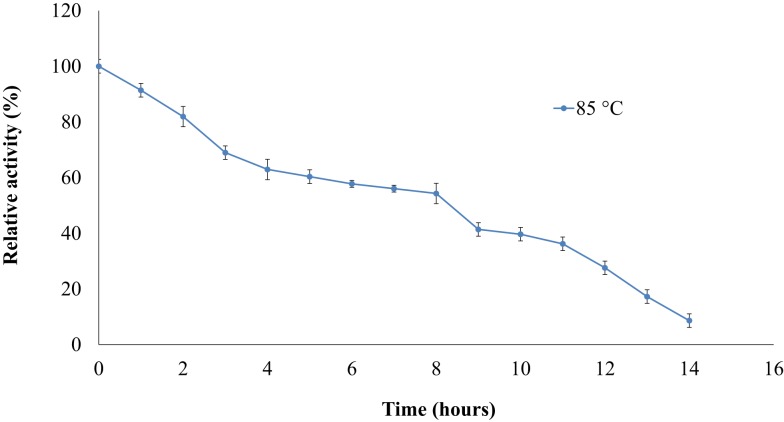
**Effect of temperature on the specific activity of NitM24D13 nitrilase as a function of time**. The activity at initial time was considered as 100% of activity and correspond to 3135. 1 U mg^−1^. The protein was maintained at 85°C. The error bars represent the SD of three independent experimental replicates.

The thermostability of the nitrilase NitM24D13 was examined. Figure 5 shows the remarkable resistance of this nitrilase to thermal inactivation. The enzyme retained more than 50% of its activity even after 8-h incubation at 85°C at an enzyme concentration of 0.035 mg ml^−1^.

### Substrate Specificity and Kinetic Behavior

**Figure 6:**
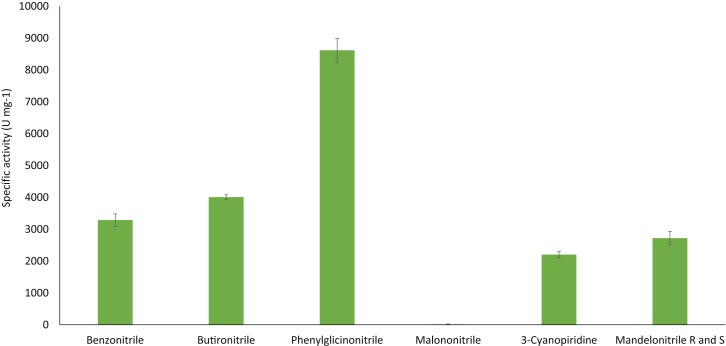
**NitM24D13 nitrilase specific activity in the presence of different substrates**. The error bars represent the SD of three independent experimental replicates.

The ability of the purified nitrilase from *Pyrococcus sp*. M24D13 to catalyze the hydrolysis of different nitriles was examined (Figure 6). The data indicate that nitrilase NitM24D13 can use a broad range of nitriles for its catalysis. There was a clear preference of the enzyme for phenylglycinonitrile and butyronitrile as substrates, suggesting that the enzyme is very versatile.

The specific activity of the purified nitrilase against benzonitrile was 3286.7 U mg^−1^ of protein. To study the affinity of the nitrilase from *Pyrococcus sp*. M24D13 toward benzonitrile, the kinetic parameters of the nitrilase were estimated over a range of benzonitrile concentration (0.5–3 mM) under standard assay condition. The maximal hydrolysis rate (*V*_máx_) and apparent Michaelis–Menten constant (*K*_m_) of nitrilase were calculated from Lineweaver Burk plot. *K*_m_ and *V*_máx_ for benzonitrile were found to be 0.3 mM and 333.3 μM min^−1^, respectively, and the specificity constant (*k*_cat_/*K*_m_) for benzonitrile was 2.05 × 10^5^ s^−1^ M^−1^.

### Effect of Metal Ions and Other Reagents

The enzyme shows high sensitivity for thiol binding metal ions such as Ag^+^, Hg^2+^. The relative activity was totally inhibited by Ag^+^ and only 2.2% of its relative activity remained in the presence of Hg^2+^. This indicates the importance of thiol group in the catalytic activity of this enzyme. The nitrilase of NitM24D13 exhibited great susceptibility to thiol-specific reagents, such as iodoacetamide (relative activity 18.5%).The presence of reducing agents, such as dithiothreitol (DTT) and glutathione, at a concentration of 5 mM, was found to inactivate the nitrilase. These data indicate the involvement of the highly conserved cysteine residue in the catalytic mechanism (Table 2).

**Table 2:** **Effect of metallic ions and inhibitors on the NitM24D13 activity**.

Inhibitor	Specific activity (U mg^**−**1^)	Residual activity (%)	Percentage of inhibition
*None*	2514.6	100.0	0
DTT 1 mM	1564.6	62.2	37.8
DTT 5 mM	0	0	100
Glutathione (reduced) 1 mM	1788.1	71.1	28.9
Glutathione (reduced) 5 mM	0	0	100.0
Iodoacetamide 1 mM	1490.1	59.3	40.7
Iodoacetamide 5 mM	4,65.7	18.5	81.5
HgCl_2_ 1 mM	260.8	10.4	89.6
HgCl_2_ 5 mM	55.9	2.2	97.8
AgNO_3_ 5 mM	0	0	100.0
KCN 1 mM	2756.7	109.6	0
KCN 5 mM	4507.6	179.3	0

EDTA and KCN had no influence on the activity of the enzyme, suggesting that the nitrilase does not need divalent ions as cofactors for catalysis. Nitrilases are not metal-dependent enzymes that are consistent with the results obtained. In the presence of 5 mM KCN, a strong increase in the specific activity of 79.3% respect to the activity measured under standard conditions was observed, suggesting that the enzyme could also have cyanidase activity (Table 2). To verify this assumption, the nitrilase activity was determined using 1 and 5 mM KCN as sole substrate, obtaining 8954.8 and 13,245.6 U mg^−1^ of activity, respectively. The activity increased 256.1% and 426.7% respect to the activity measured under standard conditions, in the presence of 3 mM benzonitrile (Table 3).

**Table 3:** **Cyanidase activity of NitM24D13 with KCN as substrate in the absence and presence of benzonitrile**.

	Specific activity (U/mg)	%
*Benzonitrile* 3 mM	2514.6	100
*Benzonitrile* 3 mM + KCN 5 mM	4507.6	179.3
*Benzonitrile* 3 mM + KCN 1 mM	2756.7	109.6
KCN 5 mM	13,245.6	526.7
KCN 1 mM	8954.8	356.1

## Discussion

The majority of currently known nitrilases from mesophilic bacterial species have been isolated from environmental samples in cultures grown using nitriles as sole nitrogen source (Layh et al., 1997). Most of them are often unstable at the temperatures optimal for growth of the microorganisms (Cowan, et al., 1998). The majority of nitrilases reported in the literature are quite labile at higher temperatures, like the *Rhodococcus rhodochrous* J1 nitrilase, which retains only 7% of its initial activity when incubated for 1 h at 50°C (Kobayashi et al., 1989). The enzymes derived from the hyperthermophilic microorganisms are more capable to do the catalysis at elevated temperatures as shown by the recombinant nitrilase from *P. abyssi* (Mueller et al., 2006). This enzyme exhibits high thermostability, after 9 h of incubation at 80°C, maintains 50% of its activity, being at 90°C the half-life of the enzyme 6 h. Due to that and to the constant demand by the industry, for new nitrilases with high stability toward temperature, substrate and product concentrations, we search for new hyperthermophilic microorganisms with potential nitrilase activity.

We found a new nitrilase that denominate NitM24D13. This enzyme has a maximum specific activity at 85°C. Thermostability of the purified nitrilase was also examined at 85°C. After 8 h of incubation at that temperature, the nitrilase NitM24D13 retains more than 50% of the specific activity. This observation indicates that the thermostability of NitM24D13 is very high and the enzyme is one of the two microbial nitrilases with highest values for activity compared to the half-life of nitrilase from *B. pallidus* Dac521 that is <3 min at 80°C (Almatawah, et al., 1999).

The majority of nitrilases described in the literature have a pH range for catalysis between 5.0 and 9.0 like nitrilases from *Alcaligenes faecalis* JM3 (Nagasawa, et al., 1990), *B. pallidus* Dac521 (Almatawah, et al., 1999), several *Rhodococcus* strains (Kobayashi, et al., 1992; Stevenson, et al., 1992), and *Pseudomonas fluorescens* DSM 7155 (Layh, et al., 1997). NitM24D13 has a tendency to catalyze the reaction at acid pH with an optimum at pH 5.5. However, the enzyme shows high activity in a wide pH range 4.5–7.5. This feature makes the enzyme suitable for the chemical industry.

The nitrilases described so far are homodimeric or multimeric, and some of them can be composed of 16 subunits (Kobayashi et al., 1990; Banerjee et al., 2002; Gong et al., 2012). Nitrilase from *Pyrococcus sp*. M24D13 was found to have a relative molecular mass of approximately 38.5 kDa as determined by size exclusion chromatography under non-denaturating conditions and the molecular mass determined in denaturing conditions was estimated as 37 kDa, which suggests that the enzyme is monomeric. Within the last few years only a few monomeric nitrilase has been described, one of them is from *R. rhodochrous* P34 and the other one is from *Arthrobacter* sp. J1 (Bandyopadhyay et al., 1986; Gong et al., 2012); for this reason, NitM24D13 is a different nitrilase to those already described enzymes. Nevertheless, it is necessary to determine the molecular mass of this protein in the presence of the substrate, by size exclusion chromatography under non-denaturating conditions to confirm if the active form of the enzyme is monomeric.

The molecular mass estimated by bioinformatic analysis of the amino acid sequence of NitM24D13 gives a mass value of approximately 30 kDa. This difference could be due to post-translational glycosylation in the microorganism increasing the molecular mass of the enzyme. It has been described the presence of glycosyltransferases in thermophilic and hyperthermophilic archaea that glycosylate proteins (Magidovich and Eichler, 2009; Calo et al., 2010). Although the N-glycosylation pathway in archaea is not yet fully understood, it is known that have certain similarities to the found in eukaryotic organisms. Oligosaccharides are transferred to the nascent protein in an asparagine residue (Asp) specifically located in an N-X-S/T motif characteristic for N-glycosylation, where X can be any amino acid except proline (Pro) (Yurist-Doutsch et al., 2008). This motif has also been identified in the amino acid sequence of NitM24D13 enzyme. Additionally, when the M24D13 genome of the microorganism was analyzed, the presence of several glycosyltransferases sequences was found. These glycosylations favor protein packaging, providing greater stability at elevated temperatures explaining the high thermostability of the enzyme at 85°C. In addition, protein glycosylation modified enzyme kinetics and decreases the effect of proteolysis on proteins (Shental-Bechor and Levy, 2008). These facts support the possibility that the enzyme NitM24D13 is glycosylated and, hence, the difference in molecular mass between the theoretical and experimental value is probably due to the presence of oligosaccharides in the protein structure. It is necessary to do further experimental studies to corroborate this assumption.

NitM24D13 nitrilase also presents a cyanidase activity by showing activity in the presence of KCN as sole substrate (Table 3), being a new hyperthermophilic enzyme that displays this type of behavior. This provides an added value to this enzyme for the biotechnological industry.

## Conflict of Interest Statement

The authors declare that the research was conducted in the absence of any commercial or financial relationships that could be construed as a potential conflict of interest.

## References

[B1] Akcil A. Karaham A. G. Ciftci H. Sagdic O. (2003). Biological treatment of cyanide by natural isolated bacteria (*Pseudomonas* sp). Miner. Eng. 16, 643–649. 10.1016/S0892-6875(03)00101-8

[B2] Almatawah Q. A. Cramp R. Cowan D. (1999). Characterization of an inducible nitrilase from a thermophilic bacillus. Extremophiles 3, 283–291. 10.1007/s007920050129 10591020

[B3] Arnaud A. Galzy P. Jallageas J. (1976). Nitrilase activity in several bacteria. C. R. Acad. Sci. Hebd. Seances Acad. Sci. 283, 571–573. 825308

[B4] Aronstein B. N. Maka A. Srivastava V. J. (1994). Chemical and biological removal of cyanides from aqueous and soil-containing systems. Appl. Biochem. Microbiol. 41, 700–707. 10.1007/BF00167288

[B5] Balch W. E. Fox C. E. Magrun L. G. Woese C. R. Wolfe R. S. (1979). Methanogens: reevaluation of a unique biological group. Microbiol. Rev. 43, 260–296. 390357 10.1128/mr.43.2.260-296.1979 PMC281474

[B6] Bandyopadhyay A. Nagasawa T. Asano Y. Fujishiro K. Tani Y. Yamada H. (1986). Purification and characterization of benzonitrilases from *Arthrobacter* sp. Strain J-1. Appl. Environ. Microbiol. 51, 302–306. 16346987 10.1128/aem.51.2.302-306.1986 PMC238863

[B7] Banerjee A. Sharma R. Banerjee U. C. (2002). The nitrile-degrading enzymes: current status and future prospects. Appl. Microbiol. Biotechnol. 60, 33–44. 10.1007/s00253-002-1062-0 12382040

[B8] Bradford M. (1976). A rapid and sensitive method for quantitation of microgram quantities of protein utilizing the principle of protein-dye-binding. Anal. Biochem. 72, 248–254. 10.1016/0003-2697(76)90527-3 942051

[B9] Calo D. Kaminski L. Eichler J. (2010). Protein glycosylation in Archaea: sweet and extreme. Glycobiology 20, 1065–1076. 10.1093/glycob/cwq055 20371512

[B10] Chauhan S. Wu S. Blumerman S. Fallon R. D. Gavagan J. E. DiCosimo R. (2003). Purification, cloning, sequencing and overexpression in *Escherichia coli* of a regioselective aliphatic nitrilase from *Acidovorax facilis* 72W. Appl. Microbiol. Biotechnol. 61, 118–122. 10.1007/s00253-002-1192-4 12655453

[B11] Cowan D. Cramp R. Pereira R. Graham D. Almatawah Q. (1998). Biochemistry and biotechnology of mesophilic and thermophilic nitrile metabolizing enzymes. Extremophiles 2, 207–216. 10.1007/s007920050062 9783167

[B12] Cramp R. Gilmour M. Cowan D. A. (1997). Novel thermophilic bacteria producing nitrile-degrading enzymes. Microbiol. 143, 2313–2320. 10.1099/00221287-143-7-2313 33657720

[B13] Daniel R. Cowan D. Morgan H. Curran M. (1982). A correlation between protein thermostability and resistance to proteolysis. Biochem. J. 207, 641–644. 6819862 10.1042/bj2070641 PMC1153914

[B14] Dash R. Balomajumder C. Kumar A. (2006). Cyanide removal by combined adsorption and biodegradation process. Iran. J. Enviro. Health. Sci. Eng. 3, 91–96.

[B15] Dash R. Gaur A. Balomajumder C. (2009). Cyanide in industrial wastewaters and its removal: A review on biotreatment. J. Haz. Mat. 163, 1–11. 10.1016/j.jhazmat.2008.06.051 18657360

[B16] DeSantis G. DiCosimo R. (2009). “Aplications of nitrile hydratases and nitrilases,” in Biocatalysis for the Pharmaceutical Industry: Discovery, Development and Manufacturing, Vol. 8, eds Tao J. Lin G.-Q. Lies A. (Singapore: John Wiley and Sons, Asia), 153–181.

[B17] Ebbs S. (2004). Biological degradation of cyanide compounds. Curr. Opin. Biotechnol. 15, 231–236. 10.1016/j.copbio.2004.03.006 15193331

[B18] Egorova K. Antranikian G. (2005). Industrial relevance of thermophilic Archaea. Curr. Opin. Microbiol. 8, 649–655. 10.1016/j.mib.2005.10.015 16257257

[B19] Ezzi-Mufaddal I. Lynch James M. (2002). Cyanide catabolizing enzymes in *Trichoderma* spp. Enzyme Microb. Technol. 31, 1042–1047. 10.1016/S0141-0229(02)00238-7

[B20] Fawcett J. K. Scott J. E. (1960). A rapid and precise method for the determination of urea. J. Clin. Pathol. 13, 156–159. 10.1136/jcp.13.2.156 13821779 PMC480024

[B21] Gong J. S. Lu Z. M. Li H. Shi J. S. Zhou Z. M. Xu Z. H. (2012). Nitrilases in nitrile biocatalysis: recent progress and forthcoming research. Microb. Cell Fact. 11, 142–159. 10.1186/1475-2859-11-142 23106943 PMC3537687

[B22] Harper D. (1985). Characterization of a nitrilase from *Nocardia* sp. (*Rhodococcus* group) NCIB 11215, using *p*-hydroxybenzonitrile as sole carbon source. Int. J. Biochem. 17, 677–683. 10.1016/0020-711X(85)90364-7 4029486

[B23] Hoyle A. J. Bunch A. W. Knowles C. J. (1998). The nitrilases of *Rhodococcus rhodochrous* NCIMB 11216. Enzyme. Microb. Technol. 23, 475–482. 10.1016/S0141-0229(98)00076-3

[B24] Jallageas A. C. Arnaud A. Galzy P. (1980). Bioconversions of nitriles and their applications. Adv. Biochem. Eng. 14, 1–32. 10.1007/BFb0007187

[B25] Kobayashi M. Nagasawa T. Yamada H. (1989). Nitrilase of *Rhodococcus rhodochrous* J1. Purification and characterization. Eur. J. Biochem. 182, 349–356. 10.1111/j.1432-1033.1989.tb14837.x 2737207

[B26] Kobayashi M. Yanaka N. Nagasawa T. Yamada H. (1990). Purification and characterization of a novel nitrilase of *Rhodococcus rhodochrous* K22 that acts on aliphatic nitriles. J. Bacteriol. 172, 4807–4815. 2394676 10.1128/jb.172.9.4807-4815.1990 PMC213134

[B27] Kobayashi M. Yanaka N. Nagasawa T. Yamada H. (1992). Primary structure of an aliphatic nitrile-degrading enzyme, aliphatic nitrilase, from *Rhodococcus rhodochrous* K22 and expression of its gene and identification of its active site residue. Biochemistry 31, 9000–9007. 10.1021/bi00152a042 1390687

[B28] Laemmli U. K. (1970). Cleavage of structural proteins during the assembly of the head of bacteriophage T4. Nature 227, 680–685. 10.1038/227680a0 5432063

[B29] Layh N. Hirrlinger B. Stolz A. Knackmuss H. J. (1997). Enrichment strategies for nitrile-hydrolysing bacteria. Appl. Microbiol. Biotechnol. 47, 668–674. 10.1007/s002530050993

[B30] Magidovich H. Eichler J. (2009). Glycosyltransferases and oligosaccharyltransferases in Archaea: putative components of the N-glycosylation pathway in the third domain of life. FEMS Microbiol. Lett. 300, 122–130. 10.1111/j.1574-6968.2009.01775.x 19765088

[B31] Mathew C. Nagasawa T. Kobayashi M. Yamada H. (1988). Nitrilase catalyzed production of nicotinic acid from 3-cyanopyridine in *Rhodococcus rhodochrous* J1. Appl. Environ. Microbiol. 54, 1030–1032. 16347598 10.1128/aem.54.4.1030-1032.1988 PMC202591

[B32] Mueller P. Egorova K. Vorgias C. E. Boutou E. Trauthwein H. Verseck S. (2006). Cloning, overexpression, and characterization of a thermoactive nitrilase from the hyperthermophilic archaeon *Pyrococcus abyssi*. Protein Expr. Purif. 47, 672–681. 10.1016/j.pep.2006.01.006 16495079

[B33] Mylerova V. Martinkova L. (2003). Synthetic application of nitrile converting enzyme. Curr. Org. Chem. 7, 1279–1295. 10.2174/1385272033486486

[B34] Nagasawa T. Mauger J. Yamada H. (1990). A novel nitrilase, arylacetonitrilase of *Alcaligenes faecalis* JM3 purification and characterization. Eur. J. Biochem. 194, 765–772. 10.1111/j.1432-1033.1990.tb19467.x 2269298

[B35] Pace H. C. Brenner C. (2001). The nitrilase superfamily: classication, structure and function. Genome Biol. 2, 1–9. 10.1186/gb-2001-2-1-reviews0001 PMC150437 11380987

[B36] Pereira R. Graham D. Rainey F. Cowan D. (1998). A novel thermostable nitrile hydratase. Extremophiles 2, 347–357. 10.1007/s007920050078 9783183

[B37] Prasad S. Misra A. Jangir V. Awasthi A. Raj J. Bhalla T. (2007). A propionitrile-induced nitrilase of *Rhodococcus* sp. NDB 1165 and its application in nicotinic acid synthesis. World J. Microbiol. Biotechnol. 23, 345–353. 10.1007/s11274-006-9230-5

[B38] Ramakrishna C. Dave H. Ravindranathan M. (1999). Microbial metabolism of nitriles and its biotechnological potential. J. Sci. Ind. Res. 58, 925–947.

[B39] Raybuck S. A. (1992). Microbes and microbial enzymes for cyanide degradation. Biodegradation 3, 3–18. 10.1007/BF00189632 1369135

[B40] Saitpu N. Nei M. (1987). The neighbor-joining method: a new method for reconstructing phylogenetic trees. Mol. Biol. Evol. 4, 406–425. 3447015 10.1093/oxfordjournals.molbev.a040454

[B41] Sammons D. W. Adams L. D. Nishizawa E. E. (1981). Ultrsensitive silver-based color staining of peptides in poliacrilamide gel electroforesis. Electrophoresis 2, 135–141. 10.1002/elps.1150020303

[B42] Shental-Bechor D. Levy Y. (2008). Effect of glycosylation on protein folding: a close look at thermodynamic stabilization. Proc. Natl. Acad. Sci. U.S.A. 105, 8256–8261. 10.1073/pnas.0801340105 18550810 PMC2448824

[B43] Stalker D. M. Malyj L. D. McBride K. E. (1988). Purification and properties of a nitrilase specific for the herbicide bromoxynil and corresponding nucleotide sequence analysis of the *bxn* gene. J. Biol. Chem. 263, 6310–6314. 2834373

[B44] Stevenson D. Feng R. Dumas F. Groleau D. Mihoc A. Storer A. (1992). Mechanistic and structural studies on *Rhodococcus* ATCC 39484 nitrilase. Biotechnol. Appl. Biochem. 15, 283–302. 1388821

[B45] Tamura K. Peterson D. Stecher G. Nei M. Kumar S. (2011). MEGA5: molecular evolutionary genetics analysis using maximun likelihood, evolutionary distance, and maximum parsimony methods. Mol. Biol. Evol. 28, 2731–2739. 10.1093/molbev/msr121 21546353 PMC3203626

[B46] Wyatt J. M. Linton E. A. (1988). “The industrial potencial of microbial nitrile biochemistry,” in Cyanide Compounds in Biology, Vol. 140 eds Evered D. Harnett S. (Chichester: Ciba foundation symposium, Wiley), 32–48. 10.1002/9780470513712.ch4 3073061

[B47] Yurist-Doutsch S. Chaban B. VanDyke D. J. Jarrell K. F. Eichler J. (2008). Sweet to the extreme: protein glycosylation in Archaea. Mol. Microbiol. 68, 1079–1084. 10.1111/j.1365-2958.2008.06224.x 18476920

